# Video observation of sharps handling and infection control practices during routine companion animal appointments

**DOI:** 10.1186/s12917-015-0503-9

**Published:** 2015-08-06

**Authors:** Maureen E. C. Anderson, J. Scott Weese

**Affiliations:** Ontario Ministry of Agriculture, Food & Rural Affairs, Guelph, ON Canada; Department of Pathobiology, University of Guelph, Guelph, ON Canada

**Keywords:** Veterinary, Companion animal, Infection control, Video observation, Cleaning, Disinfection, Sharps, Clothing, Restraint

## Abstract

**Background:**

Infection control in veterinary clinics is important for preventing pathogen spread between patients, staff and the public. There has been no direct evaluation of the use of many basic infection control practices, including sharps handling, environmental cleaning, and personal protective clothing (PPC), in companion animal clinics. The objective of this study was to describe these and other infection control practices associated with routine companion animal appointments in veterinary clinics in Ontario.

**Results:**

Video observation of practices was performed in 51 clinics for approximately 3 weeks each as part of another study evaluating the effect of a poster campaign on hand hygiene compliance. Two small wireless surveillance cameras were used: one in an exam room, one in what was considered the most likely location for hand hygiene to be performed outside the exam room following an appointment. Video footage was coded and analyzed for 47 clinics, including 2713 appointments and 4903 individual staff-animal contacts. Recapping of a needle was seen in 84 % (1137/1353) of appointments in which use was observed. Only one apparent needlestick injury (NSI) was seen, during recapping. Exam tables were cleaned and floors were mopped following 76 % (2015/2646) and 7 % (174/2643) of appointments, respectively. Contact time with spray used to clean the exam table ranged from 0–4611 s (mean 39 s, median 9 s). Appropriate PPC was worn for 72 % (3518/4903) of staff-animal contacts.

**Conclusions:**

Although there was significant room for improvement in sharps handling behaviours in participating clinics, the number of observed NSIs was low. Contact time with environmental disinfectants and use of PPC could also be improved, as well as other basic infection control practices. Education and motivation of veterinary staff to use these simple measures more effectively could potentially have a significant impact on infection control in veterinary clinics for relatively little cost.

## Background

Disease outbreaks within or originating from veterinary clinics involving patients, staff and even community members have been reported, and many others likely go unreported or unnoticed [1–4]. Close attention to appropriate infection control measures in this setting is important for preventing direct and indirect transmission of pathogens between animals, as well as transmission of zoonotic pathogens between animals and people, in order to help protect the health of patients, staff, clients and other members of the public with whom these individuals may have contact.

There is limited published research regarding the use of certain infection control measures in veterinary facilities [5–7]. Guidelines for these practices can be found in some textbooks and other publications [8–12], but their implementation has seldom been assessed. Some of these infection control measures have published research to support their effectiveness (e.g., hand hygiene, environmental cleaning) in either the human or veterinary literature [13–17], while recommendations for others are based more on known and theoretical means of pathogen transmission (e.g., use of designated clothing outside of the surgical environment that can be easily changed when contaminated) [18]. Many of these infection control practices are relatively simple to perform, but they all require varying amounts of time and effort, which can make achieving adequate compliance difficult, particularly in a busy clinic setting.

Another important part of infection control and workplace safety in any medical clinic is safe handling of sharps. The avoidance of needlestick and sharps injuries (NSIs) in human healthcare is a major priority, driven in particular by the risk of transmission of serious bloodborne pathogens between patients and healthcare workers [19, 20]. There has been less concern regarding NSIs in veterinary medicine, likely because there are currently very few recognized bloodborne pathogens that can be transmitted between domestic animals and humans. However, reports of such transmission do exist [21, 22], and the risks of other potential consequences of NSIs (e.g., trauma, local tissue infection, allergic or inflammatory reactions to drugs or biologics inoculated or injected into the tissues) are the same or potentially higher when working with animals and drugs not intended for use in humans [23–25].

The objectives of this study were to describe the following in veterinary practices in Ontario: sharps handling practices, the occurrence of observable NSIs, the use of personal protective clothing (PPC), and environmental cleaning associated with routine (non-emergency) companion animal appointments. Observations regarding various clinic-level infection control-related practices were also made.

## Methods

A convenience sample of primary care companion animal veterinary clinics from across southwestern and eastern Ontario, Canada, was recruited to participate. Briefly, clinics in various regions were identified through known contacts of either of the investigators and using Google Maps (www.maps.google.ca) with the search term “veterinary”. Each clinic was then contacted directly by one of the investigators via e-mail, fax or telephone. If no response was received, follow-up inquiries were made by the same means 1, 3 and 5 weeks later, and then monthly thereafter until recruitment was complete. Video observation in clinics was performed as part of another study evaluating the effectiveness of a poster campaign to improve hand hygiene compliance [26]. The posters, which were only present for the final 6–8 days of recording, did not include any information pertaining to non-hand hygiene infection control measures. Two wireless video surveillance cameras (Logitech WiLife^TM^ Indoor Video Security System, Logitech, Newark, CA) were installed in each clinic: one in a routinely-used exam room, and one in the most likely backroom location for hand hygiene to be performed outside the exam room following an appointment (excluding private offices and washrooms), as determined by clinic layout and information on clinic workflow provided by staff. The cameras were visible to staff but care was taken to position the cameras and secure their power cords to make them as discreet as possible. Video data were recorded by powerline network on a secure, closed laptop computer kept elsewhere in the clinic in an unobtrusive location. Cameras were left in place for 14–19 working days (19–23 calendar days), and were motion-activated during the hours when routine (non-emergency) appointments were typically scheduled in each clinic, plus approximately 30 min before and after this period. The cameras did not record audio data. Written consent was obtained from all clinic personnel whose images would potentially be captured on video; they were informed that the focus of the study was general infection control practices, but not for what specific practices data would be collected. Consent was not obtained from clients as no identifying information or information about their behaviour was collected. This study was approved by the University of Guelph Research Ethics Board.

A video coding scheme was developed in the form of a fillable spreadsheet (Excel 2008 for Mac, Microsoft Corporation, Redmond, WA). All videos were coded by the same author (MA). The appointments coded were determined by the methods for the hand hygiene compliance study [26]. Briefly, consecutive appointments were coded from the time the hand hygiene posters were placed in the clinic for a maximum of 8 days (i.e., end of recording) or up to half of the predetermined targeted maximum number of appointments. An approximately equal number of appointments was then coded working backward from the same point. The maximum number of complete appointments coded per clinic was initially set at 100, in order to maximize the amount of data that could be included from each clinic while avoiding excessive representation of very busy clinics in the data set. This target was later reduced to 80 appointments per clinic due to time constraints, the number of participating clinics and the total amount of video footage collected.

Videos were generally scanned at 2–4 times normal speed, and then watched in real time or slow motion with repeated review as necessary to discern pertinent actions. The following information was coded for each appointment: patient species (cat, dog, other, multiple); appointment type (vaccination, other); use of a sharp; ready availability of an approved sharps disposal container (i.e., labeled, puncture/leak/spill proof container not kept in a cupboard or drawer, such that sharps could be directly disposed into the container after use); uncapping of a needle using the mouth; recapping of a needle using two hands (i.e., not “scoop” technique or using an instrument); bare needle left out on any surface for any length of time; all sharps placed in a disposal container (approved or unapproved) prior to the end of the appointment; apparent NSIs; cleaning of the exam table (including contact time with spray used) and mopping of the exam room floor, respectively, within 1 h (mid-day) or 30 min (end of day) of the previous appointment; contact of a patient with the exam room floor and horizontal surface of the exam table, respectively; animal restrained by an additional staff member or client; other adverse events (e.g., scratches, bites). Contact time with table spray was measured to the second from when application of the spray began to when an individual began to wipe the table with a disposable or reusable towel. The types or brands of disinfectants used for environmental cleaning (e.g., in the table spray) were not recorded. For each staff-animal contact (coded for each unique staff member who had contact with a patient within each appointment) the use of appropriate PPC was coded, along with the individual’s gender and apparent role (veterinarian, technician, other). Appropriate PPC was defined as wearing a lab coat, scrubs or other clinic-issue uniform or clothing (i.e., bearing a clinic logo) that covered all personal clothing (i.e., street clothes) from at least the waist up other than a reasonable portion of the collar area, as well as closed-toed shoes. Lab coats worn without being buttoned or otherwise closed in the front, and scrubs or lab coats that consistently left an estimated 3–5 cm or more of the sleeve of the garment underneath exposed were not considered appropriate PPC. When observed, the type of inappropriate PPC was recorded as text in the spreadsheet. Miscellaneous noteworthy observations for each clinic pertaining to infection control were recorded separately as text.

### Statistical analysis

Coded data were imported into a statistical software package (STATA Intercooled 11, StataCorp, College Station TX) for analysis. Logistic regression, including clinic as a random effect to control for clustering in each model, was used to examine univariable associations between ready availability of an approved sharps disposal container and each of the following: recapping of a needle, a bare sharp being left out, and disposal of all sharps prior to the end of the appointment. The same type of analysis (univariable logistic regression including a random effect for clinic) was used to examine associations between cleaning of room surfaces (table and floor) and animal contact therewith. It was not possible to control for repeated measures among individuals in these models because the data was coded at the appointment level, not the individual level. Logistic regression was also used to examine the association between appropriate PPC and gender and role, respectively, including both clinic and individual as random effects. Significance was set at p ≤ 0.05. Descriptive statistics were examined for all other data.

## Results

A total of 135 clinics were approached to participate in the study, out of approximately 1100 provincially registered companion animal hospitals in Ontario (12 %). Ultimately video footage from 47 of 51 facilities where video surveillance was performed was coded and included in the analysis. Four sites were excluded from the analysis: two due to loss of data from a computer malfunction during the recording period, one due to staffing issues and plumbing problems in one of the monitored areas, and one because signs were posted by clinic staff next to the cameras alerting personnel and clients to their presence, thereby altering the conditions of the study and rendering the data not comparable to the other clinics. In 60 % (28/47) of these clinics, the monitored backroom area included at least part of a treatment area, while in the remainder it was a location where animals were generally not handled (e.g., pharmacy, lab or records area in close proximity to the monitored exam room, where hand hygiene would typically be performed between appointments).

Data from 2713 appointments were coded, including 1053 (39 %) appointments during which vaccine administration was observed and which therefore likely involved relatively healthy animals. It is possible that a number of the “other” appointments also included patient vaccination when this was performed in an unmonitored backroom area. The total number of appointments per clinic ranged from 9–104 (mean 58, median 64). A total of approximately 535 unique individuals had 4903 staff-animal contacts. The number of individuals per clinic ranged from 4–39 (mean 11, median 10), and the number of staff-animal contacts per clinic ranged from 24–251 (mean 104, median 97). The distribution of study participants by gender and role is shown in Table 1.

**Table 1:** Distribution by gender and role of study participants from 47 companion animal clinics in Ontario

Role	Number of personnel (%)		
	Male	Female	Total
Veterinarian	45 (69)	111 (24)	156 (29)
Technician	10 (15)	266 (57)	276 (52)
Other support staff^a^	10 (15)	93 (20)	103 (19)
Total	65 (100)	470 (100)	535 (100)

^a^including receptionists, students, volunteers

### Sharps handling

Thirty-six percent (17/47) of clinics had an approved sharps disposal container readily available in the exam room, but in two of these it was observed that the top of the container (i.e., the portion including the needle-removal device that also prevents accidental or intentional removal of sharps from the container) was not on tight and/or the container was periodically emptied (rather than filled, sealed and replaced). Twenty-six percent (12/47) of clinics had a sharps disposal container in a cupboard or drawer. In 30 % (14/47) of clinics there was no apparent sharps disposal container anywhere in the exam room, in which case sharps were either taken elsewhere for disposal at or after the end of the appointment, or (in 4 clinics) they were temporarily placed in an open tray or cup for disposal later in the day. In at least 2 clinics sharps brought to the backroom area were placed in an open bin rather than a disposal container. Use of inappropriate sharps containers was observed in 4 clinics: 3 used empty plastic jugs with openings of various diameters, while 1 used a metal can with a modified removable plastic lid.

Use of a hypodermic needle was observed in 50 % (1353/2713) of appointments, and use of a scalpel blade was observed in 0.3 % (8/2713), two of which also included use of a needle. Sharps handling behaviours observed and their independent associations with ready availability of an approved sharps disposal container are shown in Table 2. Disposal of all visible sharps prior to the end of the appointment was significantly associated with the availability of such a container (odds ratio (OR) = 15.00, 95 % confidence interval (CI) 6.67–33.73, *p* < 0.001). Recapping of needles using the recommended one-handed “scoop” technique [8, 11, 12, 24] was seen on four occasions (0.4 % of 1137 appointments in which a needle was recapped); in two of these cases the individual was restraining an animal with the other hand, therefore use of a one-handed technique was the only feasible way to recap the needle. Use of an instrument to recap needles was not observed, but use of an instrument to remove needles from syringes was seen once in one clinic, and was routinely used in another clinic by two individuals. Only one NSI was observed, which was sustained by a young volunteer (subjectively appearing to be less than 18 years old) recapping a needle used to administer a vaccine that had been left out on the exam room counter.

**Table 2:** Sharps handling behaviours observed during 1359 routine companion animal appointments in 47 veterinary clinics

			Independent association with ready availability of an approved sharps disposal container^a^		
Behaviour	Number of appointments (%)	Number of clinics (%)	OR	95 % CI	*p*
Uncapping of needle using the mouth	350/1353 (26)	41/47 (87)	ND	ND	ND
Recapping of needle	1137/1353 (84)	47/47 (100)	0.46	0.15–1.38	0.167
Bare sharp left out	237/1359 (17)	38/47 (81)	0.78	0.43–1.43	0.431
All visible sharps placed in disposal container prior to end of appointment	513/1359 (38)	39/47 (83)	15.00	6.67–33.73	<0.001

ND = not determined as availability of a disposal container would not be expected to affect uncapping behaviour

^a^A random effect for clinic was included in each model, and in all three models the effect was significant (p for likelihood ratio test vs logistic regression without the random effect <0.001 for all)

### Environmental cleaning

The exam table was cleaned and the exam room floor was mopped within 1 h and before the next appointment (during the day) or within 30 min (at end of day) following 76 % (2015/2646) and 7 % (174/2643) of appointments, respectively. In 7 % (178/2646) of cases the table was cleaned more than once by either the same or different staff members. All clinics used a spray to clean the table, and the contact time allowed ranged from 0 (spray applied to cleaning towel rather than directly to table) to 76 min 51 s (spray having been applied within 1 h of the appointment and then wiped off later) (mean 39 s, median 9 s, 75th percentile 15 s). Animals had contact with the exam table, the exam room floor or both in 24 % (659/2713), 28 % (748/2713) and 48 % (1295/2713) of appointments respectively. In 0.4 % (11/2713) of appointments contact of the animal with neither the table nor floor was observed (i.e., always held by owner, remained in carrier or on gurney). Table cleaning was significantly associated with patient contact with the exam table (OR = 4.69, 95 % CI 3.79–5.81, *p* < 0.001), and floor cleaning with patient contact with the exam room floor (OR = 1.53, 95 % CI 1.02–2.29, *p* = 0.040) in separate univariable models. The random effect for clinic was significant in both models (p for likelihood ratio test vs logistic regression without the random effect <0.001 for both).

### Personal protective clothing (PPC)

Appropriate PPC was worn for 72 % (3518/4903) of staff-animal contacts. Compliance with appropriate PPC within each clinic ranged from 21–99 % (mean 69 %, median 70 %). Commonly observed types of inappropriate PPC are shown in Table 3. Appropriate PPC was significantly associated with being a veterinarian (OR = 3.26, 95 % CI 1.08–9.82, p = 0.036) or technician (OR = 3.94, 95 % CI 1.37–11.33, *p* = 0.011) compared to other staff, but not with gender (*p* = 0.494). There was no significant difference between veterinarians and technicians. The random effects for clinic and individual were significant in both models (p for likelihood ratio test vs logistic regression without the random effects <0.001 for both).

**Table 3:** Inappropriate personal protective clothing (PPC) observed during 4903 veterinary staff-animal contacts in 47 veterinary clinics

Inappropriate PPC	*N* (% of total staff-animal contacts)
Long sleeves worn under short-sleeved scrubs, smock or lab coat	553 (11)
Sweater that was not clinic-issue or part of a clinic uniform worn over scrubs	316 (6)
Open lab coat over street clothes	170 (3)
Sleeves sticking out 3–5 cm or more past the cuffs of a lab coat or scrub shirt	73 (1)
Open-toed shoes	43 (1)
Street clothes alone (no PPC)	107 (2)
Other/not recorded	123 (3)

### Animal restraint

Animal restraint was recorded for 2680 appointments. In 20 % (524/2680) either no restraint was required or restraint was performed by the primary staff member conducting the appointment. In 11 % (290/2680) restraint was performed by an additional staff member, without help from the client. A client appearing to be over the age of 16 restrained (or helped restrain) the animal in 69 % (1854/2680) of appointments, and in 0.4 % (12/2680) of appointments a client appearing to be under the age of 16 helped with restraint. Restraint ranged from gentle “distraction” of the animal through physical contact to active restraint of fractious, excitable or large animals. For large dogs examined on the floor outside the field of view of the camera, it was assumed that the owner performed at least mild restraint unless there was evidence that the individual could not have done so based on position or physical limitations. Use of muzzles for both dogs and cats was observed but not coded. Only one bite was observed, which was sustained by a technician restraining a cat in the backroom. Two technicians were noted to have been scratched by cats, one while restraining and one while administering a pill. Two clients sustained scratches while restraining their own cats in the exam room; in one case the scratch was severe enough that the client was taken to the backroom to apply a bandage wrap. One other client appeared to sustain a relatively severe cat scratch (or possibly bite) immediately before entering the exam room, as evidenced by obvious bleeding and the cat’s demeanor. All observed bite and scratch injuries were to the hands.

### Other observations

Other infection control-related observations that were made on a clinic basis are shown in Table 4. It is important to note that if a particular observation was not made in a specific clinic, it does not exclude the possibility in some cases that the item/action was present/occurred elsewhere in the clinic in an unmonitored area or at an unmonitored time. These numbers therefore represent the minimum number of clinics in which the listed factors were present.

**Table 4:** Factors relating to infection control observed during video monitoring in 47 veterinary clinics

Factor	Number of clinics (%)
Paper records regularly used in exam room	37 (79)
Computer present in exam room	18 (38)
Food and/or drink consumed in clinical areas	33 (70)
Human food dishes/utensils cleaned in clinical area sink	7 (15)
Free-roaming cat(s) in clinic	25 (53)
Clinic or staff animals allowed to have contact with patients in exam room	4 (9)
Communal ear cleaner/medication bottle applied directly to ears of patients	22 (47)
Bottles of liquids (e.g., soap, antiseptic, disinfectant) “topped up”	18 (38)
Communal container or bag of treats kept/used in exam room	42 (89)
Disposable thermometer covers used	12 (26)

## Discussion

Although hand hygiene is considered by many to be the single most important measure for controlling the spread of pathogens in the clinic environment [8, 10–13], effective infection control requires diligent, every-day attention to many other practices as well. In isolation each of these practices may seem to be of limited importance, and the relative importance of most is poorly defined, but together they have the potential to help break the chain of transmission and thus reduce the incidence of infections. This study provided a unique opportunity to directly observe the use of some of these infection control practices in primary care veterinary clinics.

Attention to safe sharps handling practices is paramount to the prevention of NSIs. Sharps should be disposed immediately after use directly into an approved container (i.e., not left bare on any surface), and recapping of needles should be avoided unless no other alternative exists, as this is considered a high-risk procedure [20, 23, 27]. If an appropriate disposal container is not readily available, recapping should be done using the one-handed “scoop” technique, or using an instrument to handle the needle cap [8, 11, 12, 24]. Recapping of needles was common in this study, but recapping in the recommended manner was only seen four times, and in two of these the technique was likely only used out of necessity because the individual only had one free hand. The risk of NSIs can also be reduced by the use of safety devices (e.g., retractable needles), which are becoming more commonly used in human healthcare [19] but have yet to be widely adopted in veterinary practice. The use of needle protective devices was not observed in any clinic in this study. Bare sharps should never be left out due to the risk of injury during subsequent handling and disposal, as well as to individuals who may not realize an exposed sharp is present. Not surprisingly, the odds of disposal of all visible sharps prior to the end of an appointment were 15-times higher when an approved sharps disposal container was readily available, although the presence of such a container was not associated with the odds of staff recapping a needle or leaving a bare sharp exposed. Veterinary staff should be trained to avoid recapping needles by disposing of them directly into an approved container, and to use the “scoop” technique or an instrument for recapping when absolutely necessary. In order to facilitate this, clinics must ensure that appropriate containers are available in all areas where sharps are used. If containers are stored in a cupboard, they should be taken out and placed in the open prior to use of any sharp. The use of temporary storage or other unapproved containers for used sharps should be discouraged, as these require additional handling of used sharps during transfer to a proper disposal container, which also increases the risk of NSI [8, 11, 12]. Use of such containers in other clinics has been reported previously [23].

Uncapping of needles by mouth (i.e., gripping the needle cap in the teeth and unsheathing the needle using one hand) was also a relatively common occurrence in this study, typically when an individual only had one free hand. This behaviour results in higher risk of NSI to the face and potentially even the eyes, and should therefore be avoided at all times [8, 11, 12]. It also carries the risk of transfer to the mouth of infectious pathogens that may be present on the needle cap.

Studies have indicated that 64–93 % of veterinary personnel have experienced at least one NSI in their career [23, 25, 28–30], and in one report 74 % of veterinary technicians had experienced a NSI in the last 12 months [23]. As in human medicine, NSIs in veterinary medicine are likely significantly underreported [19, 23]. In this study only one NSI was observed among the 535 individuals whose actions were coded; however, when one considers the fraction of total activities of any one person that was captured on video and then coded, even a single NSI may represent a significant rate of occurrence given the frequency of sharps use. Furthermore, this study focused on sharps use during routine appointments, which may be lower-risk for NSIs compared to other procedures, and some NSIs may have gone undetected for various reasons, including lack of a visible physical reaction from the individual at the time. It is unknown if the volunteer who sustained the NSI observed in this study had received any training in sharps handling, which poses a potential liability issue for the clinic. Adequate training of such lay staff, restriction of sharps handling to trained professional staff and/or immediate disposal of the sharp (rather than leaving it bare on the counter) all could have potentially prevented the incident.

The role of environmental contamination in the spread of pathogens in veterinary clinics is often unclear. Studies have shown that a variety of infectious agents can persist on many different surfaces within a clinic [31–34], but in the absence of epidemiological evidence linking a certain item or surface to clinical cases, the significance of such contamination is poorly understood, with the possible exception of a few well-studied pathogens (e.g., canine parvovirus) [10]. Nonetheless, due to the potential for microbes in the environment, particularly on high-contact surfaces, to be picked up directly or indirectly by animals and people, attention to appropriate cleaning and disinfection protocols is highly recommended [8, 9, 11, 12]. The goal of these protocols is to reduce the environmental microbial burden to a level at which the risk of infection for the majority of animals and people is as low as possible. Effective disinfection first requires cleaning to remove gross contamination, followed by adequate contact time with an appropriate disinfectant that is typically then wiped or rinsed off. All clinics in this study used what appeared to be a disinfectant spray for cleaning of the exam room table and other surfaces. Recommended contact times for disinfectants typically used in veterinary clinics are 5–10 min [9, 10], and for some products may be as short as 1–3 min [35], yet in up to 75 % of observed applications, total contact time with the spray used on the exam table was 15 s or less. In these cases the surface was being cleaned, but the presumably desired low-level disinfection was not being achieved. Disinfectants must be used according to the manufacturer’s instructions for both contact time and concentration in order to be fully effective. Due to the wide variety of patients that may be seen in a veterinary clinic, and more critically the inability to detect subclinical carriers of various pathogens that may contaminate the environment even during a routine appointment, low-level disinfection of high-contact surfaces such as exam tables between all patients is recommended [8, 9, 11, 12].

A major difference between veterinary clinics and human hospitals is the degree of contact veterinary patients (and by extension the staff and clients working with the patients) have with the floor. In this study, animals had contact with the exam room floor in more appointments (76 %) than with the exam room table (72 %), yet the table was cleaned after 76 % of appointments and the floor was mopped after only 7 %. Floors were swept more often than mopped, and occasionally vacuumed, but still relatively infrequently (data not shown). Although sweeping helps to remove visible dirt and debris, including potentially contaminated hair and dander (making it an important step prior to application of a disinfectant), by itself sweeping does not reliably eliminate microbes from a surface, and may aerosolize contaminated dust or debris which may then settle on other surfaces. The same can be said for vacuums, unless a centralized unit is used and/or the vacuum is equipped with a HEPA filter [8, 12]. It is possible that this greater effort to clean exam tables over floors is for aesthetic reasons, but it is important for clinics to have a realistic view of what their current environmental cleaning practices do and do not accomplish, particularly in terms of disinfection for infection control purposes, so that protocols can be properly evaluated and adjusted if necessary.

Personal protective clothing is important for infection control within a clinic as well as for preventing spread of pathogens into the community [8, 11, 12]. Within the clinic, additional articles of clean PPC (e.g., scrubs, lab coats) appropriate in size for each staff member must be available so that individuals can change their PPC as needed when it becomes soiled or potentially contaminated. Unlike street clothes, these items are generally made from relatively durable easy-to-clean material to facilitate frequent laundering. Equally if not more importantly, PPC must not be worn outside the clinic, as infectious pathogens may be carried on clothing to anywhere the individual may go, including vehicles, eating establishments, other public places, and home, where the person may have close contact with household members and pets. In this study, no attempt was made to evaluate if and when staff changed their clothing, only whether they wore appropriate PPC during animal contacts. In at least 80 % of contacts in which PPC was classified as inappropriate, staff wore a garment that would otherwise be considered appropriate PPC but also wore an additional piece of clothing in such a way that it was not fully covered by the PPC, thus partially negating its usefulness. It is possible that these individuals wore PPC items in order to comply with clinic policy, for comfort, convenience or other reasons, without fully understanding or appreciating the rationale behind PPC, particularly in the case of staff other than veterinarians and technicians, as these individuals were less likely to wear appropriate PPC. The importance of both wearing and changing PPC as needed throughout the day and especially removing PPC at the end of the day must be emphasized to all staff.

Appropriate restraint of animals during examinations and other procedures is crucial for the safety of the individual working on the animal, the animal itself, and the person restraining. Veterinary staff in this study often relied on clients to help restrain their own animals during appointments. Although some clients can provide security and a calming influence for their pets, they may not be familiar with effective restraint techniques, and unexpected reactions from animals can lead to injuries, including bites, scratches, falls (e.g., animal falls off table) and potentially NSIs. This is a component of infection control because such trauma can lead to wound infections and transmission of some diseases (i.e., bartonellosis (cat scratch disease), rabies). All bite and scratch injuries observed in this study were caused by cats. This may be because overall cats are more likely to cause these types or injuries, because cats are harder to restrain effectively, or because staff are more cautious with dogs and therefore take additional measures to prevent injuries (e.g., muzzle, additional personnel). Even though the absolute number of bites and scratches observed was small (6), half of these were injuries to clients. As for NSIs, when one considers the fraction of clinic activity that was observed and the frequency with which the risk of such injuries is present (essentially any time an animal is handled), this may still represent a significant rate of occurrence. Injuries to clients, and even to staff if they are inadequately trained, are potentially a serious liability issue for clinics. Any time a bite or scratch occurs, the circumstances should be recorded [8, 11, 12] so that the incident can be evaluated with the appropriate staff, and staff training or clinic policies adjusted if necessary in order to reduce the risk of additional injuries. Reporting can also help ensure that appropriate wound care is exercised and that the individual is directed to seek medical attention if there is an increased risk of infection or complications (e.g., any wounds to the hands or groin, over joints or tendon sheaths, or to any individual who is immunocompromised) [12, 36].

The use of paper records in veterinary clinics remains common, even in clinics in which a computer is present in the exam room. Paper records have the potential to act as a fomite for microbes [37], are often handled by multiple individuals, and may be taken to different areas including offices where food and drink may be consumed. It was observed on numerous occasions that records were placed directly on the exam table either while the patient was still on it (in which case the animal sometimes had direct contact with the record as well) or after. Records were also frequently handled during examinations without hand hygiene being performed, thus increasing the likelihood of contamination. Staff must be aware of the potential for records to indirectly transfer microbes between people and between people and animals in order to take steps to try to reduce any such risk. The use of electronic records alone in the exam room effectively eliminates the hazard of contaminants being moved around the clinic on physical records; however, special attention must then be paid to appropriate cleaning of the computer keyboard, as this becomes a high-contact surface which can also harbor various infectious organisms [31, 32, 38].

At a clinic level, several other practices were relatively commonly noted that pose a potential increased risk to staff and/or animals from an infection control standpoint. Food and drink were seen being consumed by staff in clinical areas in 70 % of clinics. Food and drink for human consumption, including dishes and utensils used there for, should not be brought to, kept or washed in any clinical area or area where clinical samples may be placed, due to the risk of contamination and subsequent oral transmission of pathogens [8, 11, 12]. “Clinic cats” were present in over half of all clinics. The presence of (a) free-roaming animal(s) in a clinic, whether a “clinic cat” or staff-owned animal, should be strongly discouraged [12]. These animals can act as vectors or become carriers of pathogens to which they may be exposed from the environment or patients. At a minimum, such animals should not be allowed to have direct contact with patients and should be restricted from areas such as exam rooms, treatment rooms, surgical suites, and lab areas. A communal bottle of ear cleaner/medication was applied directly to the ear of patients in just under half of all clinics. This practice has the potential to result in contamination of the bottle tip (on the outside as well as the inside, which is difficult to clean) with aural pathogens, which can then potentially be transferred directly to the ears of subsequent patients. Solutions should instead be applied to disposable swabs/gauze/cotton for use on the patient, or an amount transferred to another bottle (potentially dispensed to the client) for use on a single patient. These practices should be carefully evaluated by each facility to determine if the potential benefit(s) (e.g., convenience) truly outweigh the potential increased infection risk, or if at the least an adjustment to clinic policy is warranted.

Bottles containing liquids of various kinds (e.g., soap, antiseptic, disinfectant) were seen being “topped up” in 38 % of clinics in this study. Refilling bottles without prior emptying and thorough cleaning can result in perpetual contamination with pathogenic organisms, and could potentially contribute to development of resistance to the active antiseptic or antimicrobial ingredients present [8, 9, 11, 39]. In the case of products prepared from a concentrated form, improper refilling of partially empty bottles can also result in improper dilution, thus altering the products’ effectiveness. Although it has not been formally investigated, a similar risk for perpetual bacterial contamination of containers/bags of dog and/or cat treats used during appointments exists, particularly for containers that are refilled without being emptied and properly cleaned, or for large bags that are used for a long period of time before being discarded. The majority of clinics regularly or frequently made use of such containers/bags, which were often accessed after having contact with an animal (e.g., at the end of an appointment or after a procedure) without first performing hand hygiene. Refilling of these containers was noted at times but was not formally coded.

The use of video observation in this study had both advantages and disadvantages. The ability to directly observe behaviour, rather than rely on self-reported behaviour from an interview or survey, was unique compared to previously published studies in this area. The cameras allowed for discreet observation compared to the presence of a human observer, which is still the gold standard for monitoring hand hygiene compliance in human healthcare [40]. However, the cameras were visible to staff, and all staff were made aware of the study in advance in order to provide consent, therefore Hawthorne effects could have resulted in altered (i.e., artificially improved) behaviour [40]. Based on previous work with this system, it is suspected that this effect would be relatively small [41]. Also, the appointments coded from each clinic were primarily from the latter two-thirds of the total recording period, at which point most staff would have had at least several days to become acclimatized to the presence of the cameras and resume their typical routine. Anecdotally, staff most frequently mentioned that they forgot about the cameras over time and that in the end it was “no big deal,” but on a few occasions a single staff member expressed relief when the cameras were removed. The fixed camera positions, which provided a somewhat limited and at times obstructed view, likely decreased observational sensitivity, but the level of detailed video review likely increased specificity.

Other limitations of this study should also be considered. Clinics were not randomly selected, and participated on a voluntary basis. It is possible that clinic staff with a greater interest in infection control or who were more comfortable with their current practices would be more willing to participate. This could have potentially biased the results toward better practices than the general population. Univariable models included a random effect for clinic, but did not account for clustering by individual for either sharps handling or environmental cleaning. This could have biased the results in either direction as a result of specific individuals with either particularly diligent or particularly poor practices being observed more frequently than others. However, based on the number of clinics, days, appointments and staff included in the analysis, the impact of any particular individual on the results should be minimal.

## Conclusion

Although there is significant room for improvement in sharps handling behaviours in companion animal clinics based on the frequency of high-risk behaviours observed, the incidence of perceived NSIs in this study was low. Better handling and disposal of sharps may become much more important if more zoonotic diseases emerge that are transmissible by blood contamination. Nonetheless, all veterinary personnel should be trained in safe sharps handling procedures in order to protect personnel, patients and animal owners from the risks of injury and infection, and appropriate policies should be included in the clinic infection control manual for reference.

This study suggests that there is room for improvement in veterinary clinics in the use of basic infection control measures such as environmental cleaning and use of PPC, as well as sharps handling. Improving many of these practices requires minimal financial investment, but may require time and effort on the part of personnel. The best means of achieving better compliance needs to be investigated. Increased staff training and education would be a reasonable starting point, but education alone does not necessarily result in behavioural changes. Ultimately the culture in veterinary clinics needs to include infection control as an integral part of everyday practices, through training, discussion, example and other means, so that compliance becomes automatic [42].

## Abbreviations

CI: Confidence interval
NSI: Needlestick injury
OR: Odds ratio
PPC: Personal protective clothing
